# Supplementary material for: The effects of type and workload of internal tasks on voluntary saccades in a target-distractor saccade task
Source: PLoS One. 2023 Aug 24;18(8):e0290322. doi: 10.1371/journal.pone.0290322 (PMC10449167; doi:10.1371/journal.pone.0290322)
Supplement: S1 Table — (DOCX) [file pone.0290322.s001.docx]

**S1 Table. Excluded and included trials per internal task and workload condition.**

|  | arithmetic | | | | | | visuospatial | | | | | |  |  |
| --- | --- | --- | --- | --- | --- | --- | --- | --- | --- | --- | --- | --- | --- | --- |
|  | low | | high | | control | | low | | high | | control | | Total | |
|  | n | % | n | % | n | % | n | % | n | % | n | % | n | % |
| Theoretically possible number of trials | 5000 |  | 5000 |  | 5000 |  | 5000 |  | 5000 |  | 5000 |  | 30000 |  |
| Number of trials excluded during preprocessing | 77 | 1.54 | 158 | 3.16 | 45 | 0.9 | 78 | 1.56 | 82 | 1.64 | 56 | 1.12 | 496 | 1.65 |
| Trial with more than 50% missing gaze position data | 22 | 0.44 | 29 | 0.58 | 14 | 0.28 | 6 | 0.12 | 6 | 0.12 | 14 | 0.28 | 91 | 0.30 |
| Excluded participant nr. 49 | 100 | 2 | 99 | 1.98 | 100 | 2 | 98 | 1.96 | 100 | 2 | 100 | 2 | 597 | 1.99 |
| No response to internal task | 45 | 0.9 | 147 | 2.94 | 0 | 0 | 0 | 0 | 0 | 0 | 0 | 0 | 192 | 0.64 |
| Gaze not on fixation cross | 235 | 4.7 | 316 | 6.32 | 217 | 4.34 | 287 | 5.74 | 429 | 8.58 | 244 | 4.88 | 1728 | 5.76 |
| Saccade latency <= 80ms | 76 | 1.52 | 82 | 1.64 | 57 | 1.14 | 80 | 1.6 | 99 | 1.98 | 70 | 1.4 | 464 | 1.55 |
| Saccade latency >= 600ms | 69 | 1.38 | 95 | 1.9 | 10 | 0.2 | 66 | 1.32 | 96 | 1.92 | 4 | 0.08 | 340 | 1.13 |
| Total removed trials preselection | 624 | 12.48 | 926 | 18.52 | 443 | 8.86 | 615 | 12.3 | 812 | 16.24 | 488 | 9.76 | 3908 | 13.03 |
| Total trials available after preselection | 4376 | 87.52 | 4074 | 81.48 | 4557 | 91.14 | 4385 | 87.7 | 4188 | 83.76 | 4512 | 90.24 | 26092 | 86.97 |
| Saccade landing somewhere else | 274 | 6.26 | 278 | 6.82 | 333 | 7.31 | 309 | 7.05 | 314 | 7.50 | 312 | 6.91 | 1820 | 6.98 |
| Saccade landing on distractor | 1038 | 23.72 | 850 | 20.86 | 1052 | 23.09 | 914 | 20.84 | 814 | 19.44 | 985 | 21.83 | 5653 | 21.67 |
| **Saccade landing on target** | 3064 | 70.02 | 2946 | 72.31 | 3172 | 69.61 | 3162 | 72.11 | 3060 | 73.07 | 3215 | 71.25 | 18619 | 71.36 |

Percentage values in lines 2 to 8 are relative to the theoretically possible number of trials. Percentage values in the last three lines are in relation to available trials after preselection.
