# Supplementary material for: The effects of type and workload of internal tasks on voluntary saccades in a target-distractor saccade task
Source: PLoS One. 2023 Aug 24;18(8):e0290322. doi: 10.1371/journal.pone.0290322 (PMC10449167; doi:10.1371/journal.pone.0290322)
Supplement: S4 Table — (DOCX) [file pone.0290322.s004.docx]

**Table S4. Pupil diameter TEPR: Pairwise comparisons for the interaction of task and workload per time bin.**

| Task* | Workload * | Time | Estimate | SE | DF | *t* | *p* | Effect size | BF10 | BF01 |
| --- | --- | --- | --- | --- | --- | --- | --- | --- | --- | --- |
| arithmetic | control vs. low | 0-.5 | -.02 | .00 | 87294.30 | -3.34 | .00 | -.07 | 9.15 | .01 |
|  |  | .5-1 | -.06 | .00 | 87273.97 | -11.03 | <.001 | -.24 | > 100,000 | < .01 |
|  |  | 1-1.5 | -.10 | .01 | 87193.15 | -16.05 | <.001 | -.41 | > 100,000 | < .01 |
|  |  | 1.5-2 | -.06 | .01 | 86963.58 | -7.94 | <.001 | -.25 | 1276.75 | < .01 |
|  |  | 2-2.5 | -.02 | .01 | 87016.74 | -1.78 | .23 | -.08 | .31 | 3.24 |
|  | control vs. high | 0-.5 | .01 | .00 | 87294.43 | 1.50 | .40 | .03 | 1.34 | .75 |
|  |  | .5-1 | -.01 | .00 | 8730.15 | -2.29 | .07 | -.05 | .54 | 1.86 |
|  |  | 1-1.5 | -.08 | .01 | 87298.40 | -12.22 | <.001 | -.31 | > 100,000 | < .01 |
|  |  | 1.5-2 | -.10 | .01 | 87267.70 | -13.49 | <.001 | -.42 | > 100,000 | < .01 |
|  |  | 2-2.5 | -.09 | .01 | 87294.83 | -8.71 | <.001 | -.39 | 4509.80 | < .01 |
|  | low vs. high | 0-.5 | .02 | .00 | 87291.10 | 4.81 | <.001 | .10 | > 100,000 | < .01 |
|  |  | .5-1 | .05 | .00 | 87286.88 | 8.66 | <.001 | .19 | > 100,000 | < .01 |
|  |  | 1-1.5 | .02 | .01 | 87246.43 | 3.73 | .00 | .10 | 5.26 | .19 |
|  |  | 1.5-2 | -.04 | .01 | 8728.82 | -5.58 | <.001 | -.18 | 216.08 | < .01 |
|  |  | 2-2.5 | -.07 | .01 | 87101.66 | -6.92 | <.001 | -.31 | 2114.38 | < .01 |
| visuospatial | control vs. low | 0-.5 | -.01 | .00 | 87294.35 | -1.17 | .72 | -.02 | .58 | 1.72 |
|  |  | .5-1 | -.04 | .00 | 87299.78 | -6.95 | <.001 | -.15 | 88858.21 | < .01 |
|  |  | 1-1.5 | -.08 | .01 | 8730.69 | -12.43 | <.001 | -.32 | > 100,000 | < .01 |
|  |  | 1.5-2 | -.06 | .01 | 8725.13 | -7.42 | <.001 | -.23 | 14.34 | .01 |
|  |  | 2-2.5 | -.02 | .01 | 87166.86 | -1.98 | .14 | -.09 | .35 | 2.88 |
|  | control vs. high | 0-.5 | .00 | .00 | 87294.30 | -.86 | 1.00 | -.02 | .26 | 3.83 |
|  |  | .5-1 | -.04 | .00 | 87297.87 | -7.08 | <.001 | -.16 | 57379.20 | < .01 |
|  |  | 1-1.5 | -.09 | .01 | 87274.83 | -15.20 | <.001 | -.39 | > 100,000 | < .01 |
|  |  | 1.5-2 | -.08 | .01 | 87279.40 | -11.08 | <.001 | -.35 | 4027.09 | < .01 |
|  |  | 2-2.5 | -.03 | .01 | 87259.60 | -2.63 | .03 | -.12 | .57 | 1.76 |
|  | low vs. high | 0-.5 | .00 | .00 | 8729.82 | .32 | 1.00 | .01 | .18 | 5.56 |
|  |  | .5-1 | .00 | .00 | 87293.64 | -.14 | 1.00 | .00 | .16 | 6.44 |
|  |  | 1-1.5 | -.02 | .01 | 87229.15 | -2.77 | .02 | -.07 | 1.25 | .80 |
|  |  | 1.5-2 | -.03 | .01 | 87239.75 | -3.65 | .00 | -.11 | 2.29 | .44 |
|  |  | 2-2.5 | -.01 | .01 | 87207.08 | -.65 | 1.00 | -.03 | .17 | 5.97 |
| arithmetic vs. visuospatial | control | 0-.5 | .00 | .00 | 8729.82 | -.59 | .56 | -.01 | .23 | 4.30 |
|  |  | .5-1 | .00 | .00 | 8730.91 | -.52 | .61 | -.01 | .19 | 5.23 |
|  |  | 1-1.5 | .02 | .01 | 87294.42 | 2.54 | .01 | .06 | 2.17 | .46 |
|  |  | 1.5-2 | .03 | .01 | 87288.13 | 3.71 | <.001 | .12 | 3.08 | .32 |
|  |  | 2-2.5 | .03 | .01 | 87295.48 | 2.82 | .01 | .12 | .66 | 1.50 |
|  | low | 0-.5 | .01 | .00 | 8729.89 | 1.58 | .11 | .03 | 1.50 | .67 |
|  |  | .5-1 | .02 | .00 | 8730.43 | 3.60 | <.001 | .08 | 6.44 | .16 |
|  |  | 1-1.5 | .04 | .01 | 87231.73 | 6.20 | <.001 | .16 | 172.15 | .01 |
|  |  | 1.5-2 | .03 | .01 | 87229.58 | 4.24 | <.001 | .13 | 7.14 | .14 |
|  |  | 2-2.5 | .03 | .01 | 87273.15 | 2.61 | .01 | .12 | .51 | 1.95 |
|  | high | 0-.5 | -.01 | .00 | 87291.14 | -2.94 | .00 | -.06 | 279.22 | < .01 |
|  |  | .5-1 | -.03 | .00 | 87299.94 | -5.25 | <.001 | -.12 | 1536.42 | < .01 |
|  |  | 1-1.5 | .00 | .01 | 87276.79 | -.33 | .74 | -.01 | .16 | 6.35 |
|  |  | 1.5-2 | .05 | .01 | 87298.91 | 6.19 | <.001 | .20 | 142.02 | .01 |
|  |  | 2-2.5 | .10 | .01 | 87198.46 | 8.89 | <.001 | .40 | > 100,000 | < .01 |
| arithmeticvs. visuospatial | control vs. low | 0-.5 | -.01 | .01 | 8729.86 | -1.53 | .38 | -.04 | 2.28 | .44 |
|  |  | .5-1 | -.02 | .01 | 8730.36 | -2.91 | .01 | -.09 | 5.26 | .19 |
|  |  | 1-1.5 | -.02 | .01 | 8727.00 | -2.59 | .03 | -.09 | 1.20 | .83 |
|  |  | 1.5-2 | .00 | .01 | 87128.01 | -.38 | 1.00 | -.02 | .17 | 5.86 |
|  |  | 2-2.5 | .00 | .01 | 8728.23 | .14 | 1.00 | .01 | .16 | 6.42 |
|  | control vs. high | 0-.5 | .01 | .01 | 8729.98 | 1.67 | .29 | .05 | 11.37 | .09 |
|  |  | .5-1 | .03 | .01 | 8730.84 | 3.36 | .00 | .10 | 31.03 | .03 |
|  |  | 1-1.5 | .02 | .01 | 87279.51 | 2.02 | .13 | .07 | .64 | 1.56 |
|  |  | 1.5-2 | -.02 | .01 | 87298.97 | -1.78 | .23 | -.08 | .34 | 2.97 |
|  |  | 2-2.5 | -.06 | .01 | 87276.36 | -4.33 | <.001 | -.27 | 9.24 | .11 |
|  | low vs. high | 0-.5 | .02 | .01 | 8729.97 | 3.20 | .00 | .09 | 2243.30 | < .01 |
|  |  | .5-1 | .05 | .01 | 87294.53 | 6.26 | <.001 | .20 | > 100,000 | < .01 |
|  |  | 1-1.5 | .04 | .01 | 87152.76 | 4.60 | <.001 | .17 | 54.31 | .02 |
|  |  | 1.5-2 | -.01 | .01 | 87222.01 | -1.40 | .49 | -.06 | .25 | 4.00 |
|  |  | 2-2.5 | -.07 | .01 | 87206.07 | -4.46 | <.001 | -.28 | 41.68 | .02 |

*Conditions and compared conditions, respectively. We interpreted effects if both p < .01 and BF10 >= 3. *N* = 49.
