# Supplementary material for: The effects of type and workload of internal tasks on voluntary saccades in a target-distractor saccade task
Source: PLoS One. 2023 Aug 24;18(8):e0290322. doi: 10.1371/journal.pone.0290322 (PMC10449167; doi:10.1371/journal.pone.0290322)
Supplement: S6 Table — (DOCX) [file pone.0290322.s006.docx]

**Table S6. Maintained fixation: Pairwise comparisons of workload per task.**

| Task* | Workload * | Estimate | SE | *z* | *p* | Effect size | BF10 | BF01 |
| --- | --- | --- | --- | --- | --- | --- | --- | --- |
| arithmetic | control vs. low | 0.11 | 0.10 | 1.12 | 0.787 | 0.09 | 0.36 | 2.75 |
|  | control vs. high | 0.49 | 0.09 | 5.31 | <.001 | 0.42 | 64.59 | 0.01 |
|  | low vs. high | 0.38 | 0.09 | 4.20 | <.001 | 0.32 | 40.09 | 0.03 |
| visuospatial | control vs. low | 0.20 | 0.09 | 2.17 | 0.09 | 0.17 | 0.47 | 2.11 |
|  | control vs. high | 0.67 | 0.09 | 7.87 | <.001 | 0.57 | 74.94 | 0.01 |
|  | low vs. high | 0.47 | 0.08 | 5.78 | <.001 | 0.40 | 32.67 | 0.03 |

*Conditions and compared conditions, respectively. We interpreted effects if both p < .01 and BF10 >= 3. *N* = 49.
