# Supplementary material for: The effects of type and workload of internal tasks on voluntary saccades in a target-distractor saccade task
Source: PLoS One. 2023 Aug 24;18(8):e0290322. doi: 10.1371/journal.pone.0290322 (PMC10449167; doi:10.1371/journal.pone.0290322)
Supplement: S8 Table — (DOCX) [file pone.0290322.s008.docx]

**Table S8. Correct saccade to target: Pairwise comparisons of workload per task and SOA.**

| Task* | Workload* | SOA | *z* | *p* | Cohen's *d* | BF10 | BF01 |
| --- | --- | --- | --- | --- | --- | --- | --- |
| arithmetic | control vs. low | .5 | 3.58 | 0.001 | 0.59 | 134.03 | 0.01 |
|  |  | 1 | -8.2 | <.001 | -1.45 | 761.41 | < 0.01 |
|  |  | 1.5 | 0.14 | 1 | 0.02 | 0.3 | 3.34 |
|  |  | 2 | 1.66 | 0.289 | 0.27 | 4.2 | 0.24 |
|  |  | 2.5 | 0.37 | 1 | 0.06 | 0.16 | 6.38 |
|  | control vs. high | .5 | 0.5 | 1 | 0.09 | 1.95 | 0.51 |
|  |  | 1 | -2.06 | 0.12 | -0.33 | 0.39 | 2.54 |
|  |  | 1.5 | -4.74 | <.001 | -0.84 | 0.4 | 2.5 |
|  |  | 2 | 0.88 | 1 | 0.14 | 0.6 | 1.66 |
|  |  | 2.5 | 1.19 | 0.708 | 0.2 | 0.84 | 1.18 |
|  | low vs. high | .5 | -2.94 | 0.01 | -0.5 | 0.61 | 1.64 |
|  |  | 1 | 6.1 | <.001 | 1.12 | > 100,000 | < 0.01 |
|  |  | 1.5 | -4.84 | <.001 | -0.86 | 6 | 0.17 |
|  |  | 2 | -0.76 | 1 | -0.12 | 0.21 | 4.7 |
|  |  | 2.5 | 0.82 | 1 | 0.13 | 3.18 | 0.31 |
| visuospatial | control vs. low | .5 | -3.39 | 0.002 | -0.56 | 0.34 | 2.9 |
|  |  | 1 | -0.55 | 1 | -0.09 | 0.23 | 4.34 |
|  |  | 1.5 | 1.01 | 0.941 | 0.17 | 1.34 | 0.75 |
|  |  | 2 | 0.46 | 1 | 0.07 | 0.16 | 6.4 |
|  |  | 2.5 | 2.09 | 0.11 | 0.34 | 0.52 | 1.93 |
|  | control vs. high | .5 | -4.65 | <.001 | -0.82 | 0.16 | 6.42 |
|  |  | 1 | -2.6 | 0.028 | -0.43 | 0.17 | 5.91 |
|  |  | 1.5 | 2.8 | 0.015 | 0.46 | 194.42 | < 0.01 |
|  |  | 2 | -2.53 | 0.034 | -0.41 | 0.26 | 3.85 |
|  |  | 2.5 | 1.74 | 0.247 | 0.28 | 3.06 | 0.33 |
|  | low vs. high | .5 | -1.42 | 0.47 | -0.26 | 0.26 | 3.89 |
|  |  | 1 | -2.01 | 0.132 | -0.34 | 0.2 | 4.96 |
|  |  | 1.5 | 1.78 | 0.224 | 0.3 | 10.41 | 0.1 |
|  |  | 2 | -2.96 | 0.009 | -0.48 | 0.3 | 3.38 |
|  |  | 2.5 | -0.32 | 1 | -0.05 | 0.36 | 2.76 |

*Conditions and compared conditions, respectively. We interpreted effects if both p < .01 and BF10 >= 3. *N* = 49.
