# Supplementary material for: The effects of type and workload of internal tasks on voluntary saccades in a target-distractor saccade task
Source: PLoS One. 2023 Aug 24;18(8):e0290322. doi: 10.1371/journal.pone.0290322 (PMC10449167; doi:10.1371/journal.pone.0290322)
Supplement: S9 Table — (DOCX) [file pone.0290322.s009.docx]

**S9 Table. Saccade latency: Random and fixed effects.**

| Effects | Parameter | Estimate | *SE* | *df* | *t* | *p* | *SD* | lowerCI | upperCI |
| --- | --- | --- | --- | --- | --- | --- | --- | --- | --- |
| Random effects | Participant |  |  |  |  |  | 28.65 | 23.54 | 35.32 |
|  | Trial |  |  |  |  |  | 11.99 | 7.98 | 19.28 |
|  | Residual |  |  |  |  |  | 72.73 | 71.94 | 73.41 |
| Fixed effects | (Intercept) | 260.32 | 6.29 | 65.63 | 41.39 | <.001 |  | 247.93 | 272.69 |
|  | taskvisuospatial | 2.27 | 4.07 | 18538.76 | 0.56 | 0.576 |  | -5.7 | 10.24 |
|  | loadlow | 56.17 | 4.12 | 18538.67 | 13.62 | <.001 |  | 48.08 | 64.24 |
|  | loadhigh | 85.66 | 4.14 | 18535.7 | 20.69 | <.001 |  | 77.56 | 93.78 |
|  | time1 | -4.53 | 4.2 | 18539.58 | -1.08 | 0.28 |  | -12.76 | 3.68 |
|  | time1.5 | 0.87 | 4.09 | 18539.36 | 0.21 | 0.831 |  | -7.15 | 8.88 |
|  | time2 | 1.64 | 4.17 | 18538.87 | 0.39 | 0.695 |  | -6.55 | 9.8 |
|  | time2.5 | -1.33 | 4.06 | 18537.48 | -0.33 | 0.743 |  | -9.27 | 6.61 |
|  | blockNr | -0.53 | 0.06 | 18530.92 | -8.52 | <.001 |  | -0.65 | -0.41 |
|  | taskOrder2 | -18.46 | 1.07 | 18531.89 | -17.23 | <.001 |  | -20.55 | -16.36 |
|  | taskvisuospatial:loadlow | 19.05 | 5.86 | 18539.49 | 3.25 | 0.001 |  | 7.57 | 30.54 |
|  | taskvisuospatial:loadhigh | -0.34 | 5.84 | 18536.42 | -0.06 | 0.953 |  | -11.77 | 11.08 |
|  | taskvisuospatial:time1 | 9.75 | 5.89 | 18539.57 | 1.66 | 0.098 |  | -1.78 | 21.29 |
|  | taskvisuospatial:time1.5 | -4.03 | 5.76 | 18539.21 | -0.7 | 0.484 |  | -15.31 | 7.25 |
|  | taskvisuospatial:time2 | -10.61 | 5.82 | 18538.41 | -1.82 | 0.068 |  | -22 | 0.78 |
|  | taskvisuospatial:time2.5 | -1.98 | 5.72 | 18538.68 | -0.35 | 0.729 |  | -13.19 | 9.23 |
|  | loadlow:time1 | -18.19 | 5.94 | 18539.73 | -3.06 | 0.002 |  | -29.83 | -6.54 |
|  | loadhigh:time1 | -13.12 | 5.94 | 18536.69 | -2.21 | 0.027 |  | -24.75 | -1.49 |
|  | loadlow:time1.5 | -47.82 | 5.85 | 18539.07 | -8.17 | <.001 |  | -59.26 | -36.34 |
|  | loadhigh:time1.5 | -42.92 | 5.94 | 18538.71 | -7.23 | <.001 |  | -54.56 | -31.29 |
|  | loadlow:time2 | -53.68 | 5.94 | 18536.75 | -9.04 | <.001 |  | -65.3 | -42.04 |
|  | loadhigh:time2 | -64.53 | 5.9 | 18539.35 | -10.93 | <.001 |  | -76.09 | -52.97 |
|  | loadlow:time2.5 | -34.16 | 5.82 | 18531.32 | -5.87 | <.001 |  | -45.56 | -22.76 |
|  | loadhigh:time2.5 | -61.85 | 5.84 | 18535.29 | -10.6 | <.001 |  | -73.29 | -50.43 |
|  | taskvisuospatial:loadlow:time1 | -28.48 | 8.32 | 18538.96 | -3.42 | 0.001 |  | -44.78 | -12.2 |
|  | taskvisuospatial:loadhigh:time1 | -18.39 | 8.4 | 18535.76 | -2.19 | 0.029 |  | -34.84 | -1.94 |
|  | taskvisuospatial:loadlow:time1.5 | 5.07 | 8.32 | 18536.95 | 0.61 | 0.542 |  | -11.23 | 21.35 |
|  | taskvisuospatial:loadhigh:time1.5 | -8.36 | 8.32 | 18539.74 | -1 | 0.315 |  | -24.64 | 7.95 |
|  | taskvisuospatial:loadlow:time2 | 12.55 | 8.36 | 18537.64 | 1.5 | 0.133 |  | -3.83 | 28.92 |
|  | taskvisuospatial:loadhigh:time2 | 22.91 | 8.3 | 18539.69 | 2.76 | 0.006 |  | 6.65 | 39.17 |
|  | taskvisuospatial:loadlow:time2.5 | -12.68 | 8.22 | 18539.5 | -1.54 | 0.123 |  | -28.79 | 3.42 |
|  | taskvisuospatial:loadhigh:time2.5 | 16.26 | 8.27 | 18538.48 | 1.97 | 0.049 |  | 0.08 | 32.45 |

lowerCI upperCI = lower and upper confidence interval. Parameter mes refer to the factor and the level to which the first level of this factor is compared to, e.g., loadlow stands for the factor workload and the comparison of control to low load. *N* = 49, total observations: 18,619.
