# Supplementary material for: The effects of type and workload of internal tasks on voluntary saccades in a target-distractor saccade task
Source: PLoS One. 2023 Aug 24;18(8):e0290322. doi: 10.1371/journal.pone.0290322 (PMC10449167; doi:10.1371/journal.pone.0290322)
Supplement: S10 Table — (DOCX) [file pone.0290322.s010.docx]

**Table S10. Saccade latency: Pairwise comparisons of workload and task per SOA.**

| Task* | Workload* | SOA | Estimate | SE | DF | t | p | Effect size | BF10 | BF01 |
| --- | --- | --- | --- | --- | --- | --- | --- | --- | --- | --- |
| arithmetic | control vs. low | .5 | -56.17 | 4.12 | 18538.67 | -13.62 | <.001 | -0.71 | > 100,000 | < 0.01 |
|  |  | 1 | -37.98 | 4.28 | 18538.28 | -8.88 | <.001 | -0.48 | > 100,000 | < 0.01 |
|  |  | 1.5 | -8.36 | 4.11 | 18537.92 | -2.03 | 0.127 | -0.11 | 4.02 | 0.25 |
|  |  | 2 | -2.49 | 4.23 | 18539.01 | -0.59 | 1 | -0.03 | 0.79 | 1.27 |
|  |  | 2.5 | -22 | 4.07 | 18538.86 | -5.41 | <.001 | -0.28 | 1306.73 | < 0.01 |
|  | control vs. high | .5 | -85.66 | 4.14 | 18535.7 | -20.69 | <.001 | -1.08 | > 100,000 | < 0.01 |
|  |  | 1 | -72.55 | 4.25 | 18535.16 | -17.08 | <.001 | -0.92 | > 100,000 | < 0.01 |
|  |  | 1.5 | -42.74 | 4.24 | 18538.27 | -10.09 | <.001 | -0.54 | > 100,000 | < 0.01 |
|  |  | 2 | -21.13 | 4.21 | 18539.3 | -5.02 | <.001 | -0.27 | 9558.82 | < 0.01 |
|  |  | 2.5 | -23.81 | 4.12 | 18535.27 | -5.79 | <.001 | -0.3 | 262.85 | < 0.01 |
|  | low vs. high | .5 | -29.49 | 4.16 | 18538.08 | -7.09 | <.001 | -0.37 | > 100,000 | < 0.01 |
|  |  | 1 | -34.57 | 4.31 | 18537.71 | -8.01 | <.001 | -0.44 | 18936.85 | < 0.01 |
|  |  | 1.5 | -34.39 | 4.32 | 18536.65 | -7.96 | <.001 | -0.44 | 5346 | < 0.01 |
|  |  | 2 | -18.64 | 4.22 | 18538.49 | -4.42 | <.001 | -0.24 | 102.47 | 0.01 |
|  |  | 2.5 | -1.8 | 4.19 | 18539.68 | -0.43 | 1 | -0.02 | 0.17 | 5.99 |
| visuospatial | control vs. low | .5 | -75.22 | 4.17 | 18536.17 | -18.05 | <.001 | -0.95 | > 100,000 | < 0.01 |
|  |  | 1 | -28.55 | 4.07 | 18539.38 | -7.02 | <.001 | -0.36 | 6383.87 | < 0.01 |
|  |  | 1.5 | -32.48 | 4.18 | 18517.77 | -7.77 | <.001 | -0.41 | > 100,000 | < 0.01 |
|  |  | 2 | -34.09 | 4.17 | 18537.83 | -8.17 | <.001 | -0.43 | > 100,000 | < 0.01 |
|  |  | 2.5 | -28.38 | 4.05 | 18539.54 | -7 | <.001 | -0.36 | 9598.16 | < 0.01 |
|  | control vs. high | .5 | -85.32 | 4.12 | 18538.5 | -20.72 | <.001 | -1.08 | > 100,000 | < 0.01 |
|  |  | 1 | -53.82 | 4.31 | 18538.5 | -12.48 | <.001 | -0.68 | > 100,000 | < 0.01 |
|  |  | 1.5 | -34.04 | 4.07 | 18537.14 | -8.36 | <.001 | -0.43 | > 100,000 | < 0.01 |
|  |  | 2 | -43.7 | 4.14 | 18539.72 | -10.55 | <.001 | -0.55 | > 100,000 | < 0.01 |
|  |  | 2.5 | -39.73 | 4.13 | 18539.64 | -9.62 | <.001 | -0.5 | > 100,000 | < 0.01 |
|  | low vs. high | .5 | -10.1 | 4.3 | 18539.38 | -2.35 | 0.056 | -0.13 | 3.72 | 0.27 |
|  |  | 1 | -25.27 | 4.15 | 18539.78 | -6.08 | <.001 | -0.32 | 4.49 | 0.22 |
|  |  | 1.5 | -1.57 | 4.1 | 18536.03 | -0.38 | 1 | -0.02 | 0.52 | 1.94 |
|  |  | 2 | -9.6 | 4.21 | 18536.61 | -2.28 | 0.068 | -0.12 | 0.63 | 1.58 |
|  |  | 2.5 | -11.35 | 4.13 | 18539.01 | -2.75 | 0.018 | -0.14 | 5.74 | 0.17 |
| arithmetic vs. visuospatial | Control | .5 | -2.27 | 4.07 | 18538.76 | -0.56 | 0.576 | -0.03 | 0.19 | 5.21 |
|  |  | 1 | -12.02 | 4.23 | 18538.37 | -2.84 | 0.005 | -0.15 | 1270.67 | < 0.01 |
|  |  | 1.5 | 1.76 | 4.07 | 18539.42 | 0.43 | 0.666 | 0.02 | 0.49 | 2.06 |
|  |  | 2 | 8.34 | 4.15 | 18538.96 | 2.01 | 0.044 | 0.1 | 0.19 | 5.39 |
|  |  | 2.5 | -0.29 | 4.01 | 18534.64 | -0.07 | 0.943 | 0 | 0.17 | 5.86 |
|  | low | .5 | -21.32 | 4.21 | 18539.03 | -5.06 | <.001 | -0.27 | 213.45 | < 0.01 |
|  |  | 1 | -2.59 | 4.1 | 18536.25 | -0.63 | 0.528 | -0.03 | 0.28 | 3.63 |
|  |  | 1.5 | -22.36 | 4.21 | 18532.48 | -5.31 | <.001 | -0.28 | 3.81 | 0.26 |
|  |  | 2 | -23.26 | 4.25 | 18539.34 | -5.47 | <.001 | -0.29 | 16735.63 | < 0.01 |
|  |  | 2.5 | -6.66 | 4.07 | 18538.17 | -1.64 | 0.102 | -0.08 | 0.18 | 5.54 |
|  | high | .5 | -1.93 | 4.2 | 18539.39 | -0.46 | 0.646 | -0.02 | 0.34 | 2.93 |
|  |  | 1 | 6.71 | 4.33 | 18537.09 | 1.55 | 0.122 | 0.09 | 0.41 | 2.41 |
|  |  | 1.5 | 10.46 | 4.23 | 18540.17 | 2.47 | 0.013 | 0.13 | 0.17 | 6.02 |
|  |  | 2 | -14.22 | 4.19 | 18538.63 | -3.4 | 0.001 | -0.18 | 2.82 | 0.35 |
|  |  | 2.5 | -16.21 | 4.24 | 18540.01 | -3.82 | <.001 | -0.2 | 0.88 | 1.13 |
| arithmetic vs. visuospatial | control vs. low | .5 | 19.05 | 5.86 | 18539.49 | 3.25 | 0.003 | 0.24 | 177.89 | 0.01 |
|  |  | 1 | -9.43 | 5.89 | 18537.29 | -1.6 | 0.328 | -0.12 | 0.78 | 1.28 |
|  |  | 1.5 | 24.12 | 5.88 | 18527.69 | 4.1 | <.001 | 0.3 | 52.37 | 0.02 |
|  |  | 2 | 31.6 | 5.95 | 18537.37 | 5.31 | <.001 | 0.4 | 8401.51 | < 0.01 |
|  |  | 2.5 | 6.37 | 5.71 | 18536.52 | 1.12 | 0.793 | 0.08 | 0.22 | 4.64 |
|  | control vs. high | .5 | -0.34 | 5.84 | 18536.42 | -0.06 | 1 | 0 | 0.5 | 1.99 |
|  |  | 1 | -18.73 | 6.05 | 18536.43 | -3.1 | 0.006 | -0.24 | 127.39 | 0.01 |
|  |  | 1.5 | -8.7 | 5.88 | 18539.12 | -1.48 | 0.418 | -0.11 | 0.19 | 5.23 |
|  |  | 2 | 22.57 | 5.9 | 18537.62 | 3.82 | <.001 | 0.28 | 6.27 | 0.16 |
|  |  | 2.5 | 15.92 | 5.83 | 18537.42 | 2.73 | 0.019 | 0.2 | 0.64 | 1.57 |
|  | low vs. high | .5 | -19.39 | 5.97 | 18539.85 | -3.25 | 0.004 | -0.24 | 19416.99 | < 0.01 |
|  |  | 1 | -9.3 | 5.98 | 18538.89 | -1.55 | 0.36 | -0.12 | 2.28 | 0.44 |
|  |  | 1.5 | -32.82 | 5.98 | 18537.73 | -5.48 | <.001 | -0.42 | 8.26 | 0.12 |
|  |  | 2 | -9.04 | 5.96 | 18537.33 | -1.51 | 0.39 | -0.11 | 1.48 | 0.68 |
|  |  | 2.5 | 9.55 | 5.88 | 18539.53 | 1.62 | 0.313 | 0.12 | 0.31 | 3.2 |

*Conditions and compared conditions, respectively. We interpreted effects if both p < .01 and BF10 >= 3. *N* = 49.
