# Supplementary material for: The effects of type and workload of internal tasks on voluntary saccades in a target-distractor saccade task
Source: PLoS One. 2023 Aug 24;18(8):e0290322. doi: 10.1371/journal.pone.0290322 (PMC10449167; doi:10.1371/journal.pone.0290322)
Supplement: S13 Table — (DOCX) [file pone.0290322.s013.docx]

**Table S13. Blinks: Pairwise comparisons of workload per task and time bin.**

| Task | Workload Comparison | Time | Estimate | *SE* | *z* | *p* | Effect size | BF10 | BF01 |
| --- | --- | --- | --- | --- | --- | --- | --- | --- | --- |
| arithmetic | control vs. low | 0-.5 | 0.19 | 0.07 | 2.61 | 0.027 | 0.14 | 0.45 | 2.2 |
|  |  | .5-1 | -0.23 | 0.08 | -2.8 | 0.016 | -0.16 | 0.52 | 1.92 |
|  |  | 1-1.5 | -0.7 | 0.12 | -6.06 | <.001 | -0.5 | 35.9 | 0.03 |
|  |  | 1.5-2 | -0.48 | 0.14 | -3.42 | 0.002 | -0.34 | 21.76 | 0.05 |
|  |  | 2-2.5 | -0.02 | 0.19 | -0.1 | 1 | -0.01 | 0.16 | 6.44 |
|  | control vs. high | 0-.5 | -0.01 | 0.07 | -0.18 | 1 | -0.01 | 0.16 | 6.43 |
|  |  | .5-1 | -0.04 | 0.09 | -0.53 | 1 | -0.03 | 0.16 | 6.26 |
|  |  | 1-1.5 | -0.79 | 0.12 | -6.86 | <.001 | -0.57 | 49.41 | 0.02 |
|  |  | 1.5-2 | -0.72 | 0.14 | -5.33 | <.001 | -0.52 | 61.2 | 0.02 |
|  |  | 2-2.5 | -0.22 | 0.18 | -1.21 | 0.678 | -0.16 | 0.34 | 2.96 |
|  | low vs. high | 0-.5 | -0.21 | 0.07 | -2.76 | 0.017 | -0.15 | 1.27 | 0.79 |
|  |  | .5-1 | 0.18 | 0.08 | 2.24 | 0.075 | 0.13 | 0.96 | 1.04 |
|  |  | 1-1.5 | -0.09 | 0.1 | -0.9 | 1 | -0.06 | 0.17 | 5.96 |
|  |  | 1.5-2 | -0.24 | 0.12 | -2.01 | 0.133 | -0.18 | 0.54 | 1.86 |
|  |  | 2-2.5 | -0.2 | 0.18 | -1.11 | 0.806 | -0.14 | 0.34 | 2.92 |
| visuospatial | control vs. low | 0-.5 | 0.1 | 0.07 | 1.39 | 0.496 | 0.07 | 0.19 | 5.25 |
|  |  | .5-1 | -0.31 | 0.08 | -3.87 | <.001 | -0.23 | 1.94 | 0.52 |
|  |  | 1-1.5 | -0.67 | 0.11 | -5.96 | <.001 | -0.48 | 9.97 | 0.1 |
|  |  | 1.5-2 | -0.38 | 0.14 | -2.72 | 0.02 | -0.28 | 0.66 | 1.51 |
|  |  | 2-2.5 | -0.23 | 0.21 | -1.08 | 0.839 | -0.16 | 0.18 | 5.39 |
|  | control vs. high | 0-.5 | 0.14 | 0.07 | 2.04 | 0.124 | 0.1 | 0.24 | 4.11 |
|  |  | .5-1 | -0.42 | 0.08 | -5.21 | <.001 | -0.3 | 18.83 | 0.05 |
|  |  | 1-1.5 | -0.72 | 0.11 | -6.52 | <.001 | -0.52 | 21.67 | 0.05 |
|  |  | 1.5-2 | -0.61 | 0.14 | -4.45 | <.001 | -0.44 | 2.6 | 0.38 |
|  |  | 2-2.5 | -0.49 | 0.2 | -2.47 | 0.04 | -0.36 | 2.99 | 0.34 |
|  | low vs. high | 0-.5 | 0.05 | 0.07 | 0.66 | 1 | 0.03 | 0.17 | 5.73 |
|  |  | .5-1 | -0.1 | 0.08 | -1.36 | 0.518 | -0.07 | 0.46 | 2.2 |
|  |  | 1-1.5 | -0.06 | 0.1 | -0.59 | 1 | -0.04 | 0.21 | 4.7 |
|  |  | 1.5-2 | -0.22 | 0.13 | -1.77 | 0.232 | -0.16 | 1.01 | 0.99 |
|  |  | 2-2.5 | -0.27 | 0.19 | -1.38 | 0.505 | -0.19 | 0.45 | 2.21 |

We interpreted effects if both p < .01 and BF10 >= 3. *N* = 49.
