# Supplementary material for: The effects of type and workload of internal tasks on voluntary saccades in a target-distractor saccade task
Source: PLoS One. 2023 Aug 24;18(8):e0290322. doi: 10.1371/journal.pone.0290322 (PMC10449167; doi:10.1371/journal.pone.0290322)
Supplement: S14 Table — (DOCX) [file pone.0290322.s014.docx]

**S14 Table. Saccades before saccade target: Random and fixed effects.**

| Effects | Parameter | Estimate | *SE* | *z* | *p* | *SD* | lowerCI | upperCI |
| --- | --- | --- | --- | --- | --- | --- | --- | --- |
| Random effects | Participant |  |  |  |  | 1.08 |  |  |
|  | Trial |  |  |  |  | 0.12 |  |  |
| Fixed effects | (Intercept) | -2.06 | 0.16 | -12.55 | <.001 |  | -2.38 | -1.74 |
|  | taskvisuospatial | 0.16 | 0.06 | 2.83 | 0.005 |  | 0.05 | 0.27 |
|  | loadlow | -0.08 | 0.06 | -1.35 | 0.177 |  | -0.2 | 0.04 |
|  | loadhigh | 0.16 | 0.06 | 2.71 | 0.007 |  | 0.04 | 0.27 |
|  | time0.5-1 | 0.14 | 0.06 | 2.28 | 0.023 |  | 0.02 | 0.26 |
|  | time1-1.5 | 0.18 | 0.07 | 2.69 | 0.007 |  | 0.05 | 0.3 |
|  | time1.5-2 | 0.2 | 0.07 | 2.65 | 0.008 |  | 0.05 | 0.34 |
|  | time2-2.5 | 0.06 | 0.1 | 0.62 | 0.533 |  | -0.13 | 0.26 |
|  | taskvisuospatial:loadlow | 0.15 | 0.08 | 1.83 | 0.068 |  | -0.01 | 0.31 |
|  | taskvisuospatial:loadhigh | -0.12 | 0.08 | -1.51 | 0.131 |  | -0.28 | 0.04 |
|  | taskvisuospatial:time0.5-1 | -0.08 | 0.09 | -0.99 | 0.324 |  | -0.25 | 0.08 |
|  | taskvisuospatial:time1-1.5 | -0.04 | 0.09 | -0.47 | 0.64 |  | -0.22 | 0.14 |
|  | taskvisuospatial:time1.5-2 | -0.11 | 0.1 | -1.02 | 0.305 |  | -0.31 | 0.1 |
|  | taskvisuospatial:time2-2.5 | 0.18 | 0.14 | 1.3 | 0.193 |  | -0.09 | 0.44 |
|  | loadlow:time0.5-1 | 0.15 | 0.09 | 1.69 | 0.09 |  | -0.02 | 0.32 |
|  | loadhigh:time0.5-1 | -0.01 | 0.09 | -0.16 | 0.877 |  | -0.18 | 0.15 |
|  | loadlow:time1-1.5 | 0.51 | 0.09 | 5.62 | <.001 |  | 0.33 | 0.69 |
|  | loadhigh:time1-1.5 | 0.2 | 0.09 | 2.15 | 0.031 |  | 0.02 | 0.37 |
|  | loadlow:time1.5-2 | 0.44 | 0.1 | 4.25 | <.001 |  | 0.24 | 0.64 |
|  | loadhigh:time1.5-2 | 0.28 | 0.1 | 2.79 | 0.005 |  | 0.09 | 0.49 |
|  | loadlow:time2-2.5 | 0.18 | 0.14 | 1.28 | 0.201 |  | -0.1 | 0.46 |
|  | loadhigh:time2-2.5 | 0.37 | 0.14 | 2.75 | 0.006 |  | 0.11 | 0.64 |
|  | taskvisuospatial:loadlow:time0.5-1 | 0.35 | 0.12 | 2.93 | 0.003 |  | 0.12 | 0.58 |
|  | taskvisuospatial:loadhigh:time0.5-1 | 0.62 | 0.12 | 5.25 | <.001 |  | 0.38 | 0.84 |
|  | taskvisuospatial:loadlow:time1-1.5 | -0.05 | 0.12 | -0.42 | 0.675 |  | -0.3 | 0.19 |
|  | taskvisuospatial:loadhigh:time1-1.5 | 0.41 | 0.12 | 3.25 | 0.001 |  | 0.16 | 0.65 |
|  | taskvisuospatial:loadlow:time1.5-2 | -0.04 | 0.14 | -0.28 | 0.777 |  | -0.32 | 0.24 |
|  | taskvisuospatial:loadhigh:time1.5-2 | 0.25 | 0.14 | 1.77 | 0.077 |  | -0.03 | 0.53 |
|  | taskvisuospatial:loadlow:time2-2.5 | -0.06 | 0.19 | -0.31 | 0.757 |  | -0.43 | 0.31 |
|  | taskvisuospatial:loadhigh:time2-2.5 | -0.13 | 0.18 | -0.71 | 0.477 |  | -0.5 | 0.23 |

Parameter mes refer to the factor and the level to which the first level of this factor is compared to, e.g., loadlow stands for the factor workload and the comparison of control to low load. lowerCI, upperCI = confidence intervals, Note: confidence intervals in this analyses were estimated with the less accurate Wald-method since the profile-method did not reach a result within 2 days. *N* = 49, total observations: 87,560.
