# Supplementary material for: The effects of type and workload of internal tasks on voluntary saccades in a target-distractor saccade task
Source: PLoS One. 2023 Aug 24;18(8):e0290322. doi: 10.1371/journal.pone.0290322 (PMC10449167; doi:10.1371/journal.pone.0290322)
Supplement: S15 Table — (DOCX) [file pone.0290322.s015.docx]

**S15 Table. Saccades before saccade target: Pairwise comparisons of workload and task per time Bin.**

| Task* | Workload* | Time | Estimate | SE | z | p | Effect size | BF10 | BF01 |
| --- | --- | --- | --- | --- | --- | --- | --- | --- | --- |
| arithmetic | control vs. low | 0-.5 | 0.08 | 0.06 | 1.35 | 0.532 | 0.07 | 0.24 | 4.08 |
|  |  | .5-1 | -0.07 | 0.06 | -1.05 | 0.878 | -0.06 | 0.17 | 5.82 |
|  |  | 1-1.5 | -0.43 | 0.07 | -6.23 | <.001 | -0.4 | 39.18 | 0.03 |
|  |  | 1.5-2 | -0.36 | 0.09 | -4.24 | <.001 | -0.33 | 29.16 | 0.03 |
|  |  | 2-2.5 | -0.1 | 0.13 | -0.78 | 1 | -0.09 | 0.22 | 4.53 |
|  | control vs. high | 0-.5 | -0.16 | 0.06 | -2.71 | 0.02 | -0.14 | 0.53 | 1.89 |
|  |  | .5-1 | -0.14 | 0.06 | -2.29 | 0.066 | -0.13 | 0.24 | 4.1 |
|  |  | 1-1.5 | -0.35 | 0.07 | -5.02 | <.001 | -0.32 | 5.3 | 0.19 |
|  |  | 1.5-2 | -0.44 | 0.09 | -5.21 | <.001 | -0.41 | 44.7 | 0.02 |
|  |  | 2-2.5 | -0.53 | 0.12 | -4.3 | <.001 | -0.48 | 271.58 | < 0.01 |
|  | low vs. high | 0-.5 | -0.24 | 0.06 | -4.01 | <.001 | -0.22 | 26.6 | 0.04 |
|  |  | .5-1 | -0.08 | 0.06 | -1.23 | 0.653 | -0.07 | 0.31 | 3.21 |
|  |  | 1-1.5 | 0.08 | 0.07 | 1.2 | 0.693 | 0.07 | 0.2 | 5.11 |
|  |  | 1.5-2 | -0.08 | 0.08 | -1 | 0.95 | -0.07 | 0.2 | 5.1 |
|  |  | 2-2.5 | -0.43 | 0.12 | -3.53 | 0.001 | -0.39 | 40.76 | 0.03 |
| visuospatial | control vs. low | 0-.5 | -0.07 | 0.06 | -1.22 | 0.663 | -0.06 | 0.21 | 4.86 |
|  |  | .5-1 | -0.56 | 0.06 | -9.57 | <.001 | -0.52 | 647.82 | < 0.01 |
|  |  | 1-1.5 | -0.53 | 0.07 | -7.92 | <.001 | -0.48 | 5334.34 | < 0.01 |
|  |  | 1.5-2 | -0.47 | 0.08 | -5.64 | <.001 | -0.43 | 177.4 | 0.01 |
|  |  | 2-2.5 | -0.19 | 0.12 | -1.62 | 0.314 | -0.17 | 0.44 | 2.27 |
|  | control vs. high | 0-.5 | -0.04 | 0.06 | -0.64 | 1 | -0.03 | 0.16 | 6.05 |
|  |  | .5-1 | -0.64 | 0.06 | -10.92 | <.001 | -0.58 | 366.17 | < 0.01 |
|  |  | 1-1.5 | -0.64 | 0.07 | -9.6 | <.001 | -0.58 | 69614.92 | < 0.01 |
|  |  | 1.5-2 | -0.57 | 0.08 | -6.95 | <.001 | -0.52 | 740.71 | < 0.01 |
|  |  | 2-2.5 | -0.28 | 0.12 | -2.39 | 0.051 | -0.25 | 0.47 | 2.12 |
|  | low vs. high | 0-.5 | 0.03 | 0.06 | 0.59 | 1 | 0.03 | 0.17 | 5.72 |
|  |  | .5-1 | -0.07 | 0.06 | -1.38 | 0.505 | -0.07 | 0.31 | 3.2 |
|  |  | 1-1.5 | -0.11 | 0.06 | -1.73 | 0.252 | -0.1 | 0.53 | 1.89 |
|  |  | 1.5-2 | -0.1 | 0.08 | -1.33 | 0.548 | -0.1 | 0.29 | 3.46 |
|  |  | 2-2.5 | -0.09 | 0.11 | -0.77 | 1 | -0.08 | 0.18 | 5.49 |

*Conditions and compared conditions, respectively. We interpreted effects if both p < .01 and BF10 >= 3. *N* = 49.
