# Supplementary figures and images for: The effects of type and workload of internal tasks on voluntary saccades in a target-distractor saccade task
Source: PLoS One. 2023 Aug 24;18(8):e0290322. doi: 10.1371/journal.pone.0290322 (PMC10449167; doi:10.1371/journal.pone.0290322)

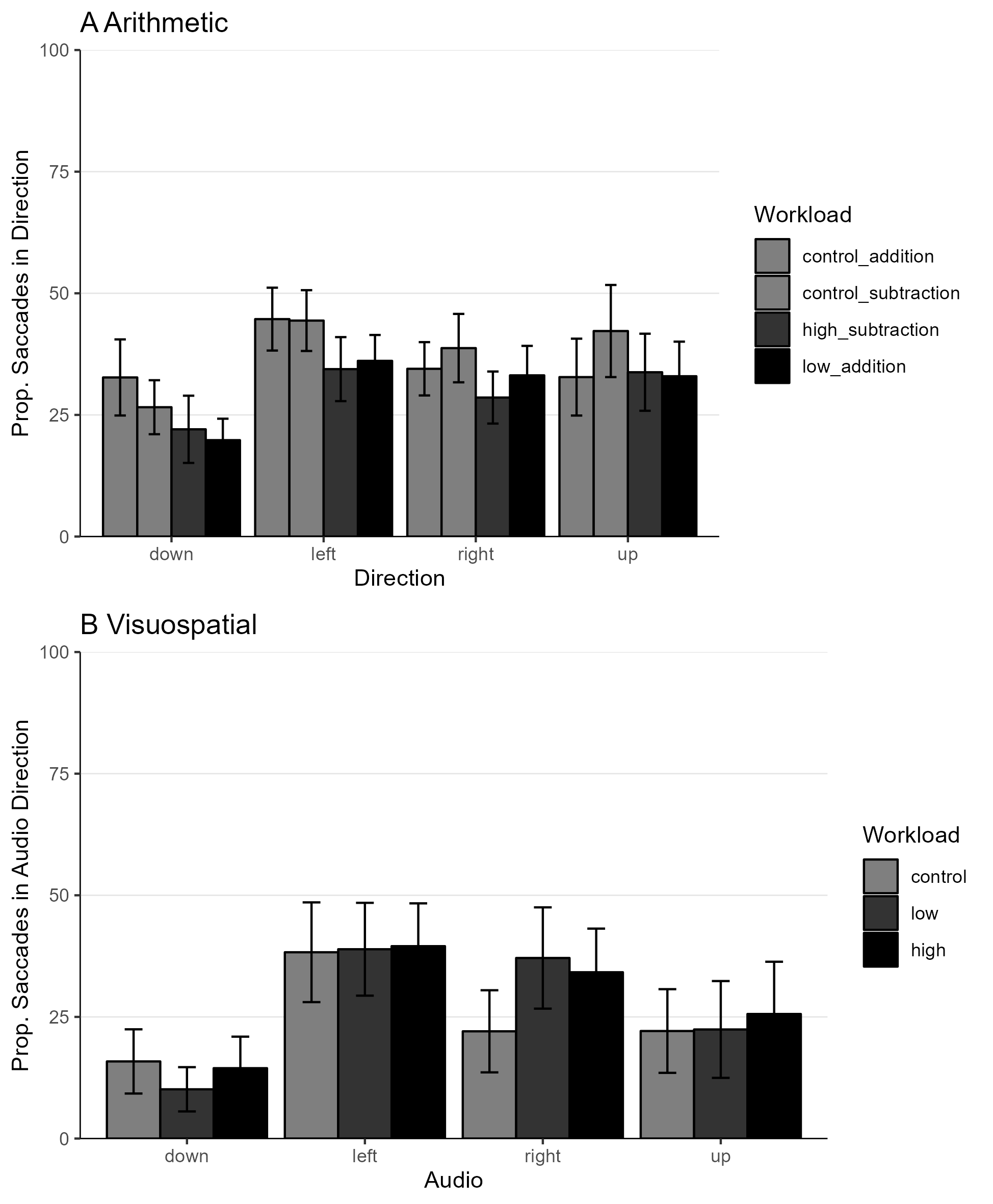

Supplement: S1 Fig — (A) Effect of the arithmetic task on the direction of pre-target saccades. X-axis shows the principal direction of the saccade, y-axis the proportion of all pre-target saccades that were in this direction, and color whether it was addition or subtraction and whether it was control or internal task condition (low, high workload). (B) Proportion of pre-target saccades that were made in the direction of the audio command. X-axis shows the direction that was presented via headphones, y-axis shows the proportion of all pre-target saccades that were made in the direction of the audio, and color shows the workload condition (control, low, high). Please note that number of pre-target saccades was overall low. (TIF) [file pone.0290322.s017.tif]
