# Supplementary material for: The effects of type and workload of internal tasks on voluntary saccades in a target-distractor saccade task
Source: PLoS One. 2023 Aug 24;18(8):e0290322. doi: 10.1371/journal.pone.0290322 (PMC10449167; doi:10.1371/journal.pone.0290322)
Supplement: Attachment — Submitted filename: Response to Reviewers.docx [file pone.0290322.s018.docx]

**Response to Reviewers**

Manuscript: PONE-D-23-07956R1

Reviewer #3: Many thanks for carefully addressing all my concerns. Eventually, I only have a very minor request related to the binomial GLMM for task performance. Here, the grouping structure of the data with the blocks is unclear to me. It sounds like you calculated a summary statistic (like mean performance across a block) and this statistic was your base level for the statistical model, but this approach would be incompatible with a binomial model. So, I guess you did indeed take individual trial data. It surprises me that it was that hard to get the model to converge. In such a case and with that many correct trials, I would consequently also rather not draw too strong conclusions from the ANOVA results and recommend to add a short paragraph to the results section that describes your logic for why you chose the ANOVA.

**Response**: Thank you for pointing out, that it was not clear in the manuscript that the performance was measured at the end of a block, hence after 9-11 trials of audio directions/numbers (see Figure 1) and not after each trial. We therefore used this binomial measure of performance per block (not trial) as input for the binomial GLMM of performance. We now extended the note below Figure 1 with the info in the brackets: “*At the end of each block, participants were asked to report the result of the internal task (this report is used as the performance measure)*.” And also in the methods section we included brackets, e.g., in line 238: “*At the end of each arithmetic task block (after 9-11 trials), participants had to type in the result*.”

Further, in the analysis strategy and results section, we also mention more clearly why there is a measure per block and not per trial for performance analysis:

Note of Figure 2, added brackets: “*The proportion of blocks with correct responses in the internal tasks (participants had to report the final result after 9-11 trials at the end of a block, see Fig 1).*”

In the methods section, at “Analysis strategy”,:

“*For internal task performance, we calculated binomial GLMMs, using the response per block (correct, incorrect). Participants reported the result of the arithmetic task, and the final position of the patch in the matrix, respectively, at the end of a block, hence after 9-11 trials, leading to one performance measure per block, not trial, see Fig 1.”*

And we now include in the results section what we previously only included in our response to your comments in the last round of revision:

“*We tried to calculate binomial GLMMs for internal task performance, using the response per block (correct, incorrect). (Participants reported the result of the arithmetic task, and the final position of the patch in the matrix, respectively, at the end of a block, hence after 9-11 trials, leading to one performance measure per block, not trial, see Fig 1.). However, the models did not converge and had singularity. We therefore reverted to a repeated-measures ANOVA using the proportion of correct blocks per Task and Workload.”*

Since we had block data instead of trial data for the binomial GLMM of performance, the overall number of observations is much lower for this analysis, potentially explaining why these GLMMs did not converge.
